# Coronary Microvascular Disease in Chronic Chagas Cardiomyopathy Including an Overview on History, Pathology, and Other Proposed Pathogenic Mechanisms

**DOI:** 10.1371/journal.pntd.0000674

**Published:** 2010-08-31

**Authors:** Marcos A. Rossi, Herbert B. Tanowitz, Lygia M. Malvestio, Mara R. Celes, Erica C. Campos, Valdecir Blefari, Cibele M. Prado

**Affiliations:** 1 Department of Pathology, Laboratory of Cellular and Molecular Cardiology, Faculty of Medicine of Ribeirão Preto, University of São Paulo, Ribeirão Preto, São Paulo, Brazil; 2 Department of Pathology, Albert Einstein College of Medicine, Bronx, New York, United States of America; London School of Hygiene & Tropical Medicine, United Kingdom

## Abstract

This review focuses on the short and bewildered history of Brazilian scientist Carlos Chagas's discovery and subsequent developments, the anatomopathological features of chronic Chagas cardiomyopathy (CCC), an overview on the controversies surrounding theories concerning its pathogenesis, and studies that support the microvascular hypothesis to further explain the pathological features and clinical course of CCC. It is our belief that knowledge of this particular and remarkable cardiomyopathy will shed light not only on the microvascular involvement of its pathogenesis, but also on the pathogenetic processes of other cardiomyopathies, which will hopefully provide a better understanding of the various changes that may lead to an end-stage heart disease with similar features. This review is written to celebrate the 100th anniversary of the discovery of Chagas disease.

## Introduction

Abnormalities of microcirculation have been demonstrated in several different cardiomyopathies [1], [2], and microvascular spasm was proposed as a common pathogenic mechanism for the development of the characteristic focal myocytolytic necrosis in these cardiomyopathies [3]–[6].

We suggested that alterations in the microvasculature contributed to the pathogenesis of experimental chronic Chagas cardiomyopathy (CCC) [7], [8]. Mice infected with *Trypanosoma cruzi* (*T. cruzi*) developed a chronic cardiomyopathy similar to that observed in the chronic phase of Chagas disease in humans. Aggregated platelets forming transient occlusive thrombi were found in small epicardial and intramyocardial vessels associated with foci of myocytolytic necrosis and degeneration with an inflammatory mononuclear infiltrate and interstitial fibrosis. Soon afterwards, areas of focal vascular constrictions, microaneurysm formation, and dilatation were demonstrated in mice acutely infected with *T. cruzi* [9]. In 1990, we proposed the participation of microcirculation via transient ischemia in the pathogenesis of CCC [10]. At that time, two hypotheses regarding the pathogenesis of CCC were being intensely investigated: parasympathetic intrinsic denervation as the mechanism of cardioneuropathy [11]–[13] and the participation of autoimmune mechanisms in the genesis of chronic fibrosing myocarditis [14]–[16].

This review focuses on the short and bewildered history of Chagas's discovery and subsequent developments, the anatomopathological features of CCC, an overview of the controversies surrounding theories concerning the pathogenesis of CCC, and studies that support the microvascular hypothesis that further explains the pathology and clinical course of CCC. It is our belief that knowledge of this particular and remarkable cardiomyopathy will shed light not only on the microvascular involvement of its pathogenesis, but also on the pathogenetic processes of other cardiomyopathies, which will hopefully provide a better understanding of the various changes that may lead to an end-stage heart disease with similar features. As stressed by Factor [17], “… the similarity of Chagas disease to other dilated congestive cardiomyopathies, particularly those due to viral etiology, should make awareness of the South and Central American disease relevant to investigators outside endemic areas”. Moreover, as a consequence of increased global migration due to socioeconomic reasons and facilitated by international travel, Chagas disease may expand exponentially from rural and endemic areas to urban and nonendemic areas, respectively. Furthermore, this review marks the 100th anniversary of the discovery of the disease by the Brazilian scientist Carlos Chagas. Simultaneously, 2009 marked a hundred years of negligence concerning Chagas disease, which is endemic in the most impoverished populations in Latin America that are still living in poor quality housing with substandard conditions, i.e., the primary habitat for *T. cruzi* vectors and mode of transmission besides blood transfusion, oral, and congenital *T. cruzi* infection transmission.

## Carlos Chagas's Discovery

In 1908, the Brazilian government, when building a railroad from Rio de Janeiro (the capital of Brazil at the time) to Belem (in the north of the Amazon Basin), a task that was never completed, had to halt construction in Minas Gerais, not too far from Rio de Janeiro, because of a severe malaria outbreak involving the railroad workers [11], [18], [19]. Oswaldo Cruz, director of Manguinhos Institute in Rio de Janeiro (currently known as Oswaldo Cruz Institute), commissioned Carlos Chagas and Belisario Pena to that region in an attempt to control the outbreak. They settled their headquarters in Lassance in a railroad car, which served as the consultation room, laboratory, and quarters. After one year of intensive work, Chagas was told by a railroad engineer about the existence of hematophagous bugs, which were known as “barbeiros” (barbers) or “kissing bugs” due to their typical behavior of biting sleeping human beings at night on the uncovered face. Chagas became interested in investigating the possibility of this bug transmitting parasites to humans or other vertebrates. He soon detected flagellates resembling crithidiae in the bugs' hindgut. Intrigued by the possibility that this parasite could represent an evolutionary stage of *Trypanosoma minasense*, which he had previously described infesting marmosets, he sent some bugs to Manguinhos to be fed to primates that were free of infection. After some weeks, the same flagellates seen in the hindgut of the bugs were recovered from the bloodstream of the animals, and a new species different from *T. minasense* or “any other species of the same genus” was recognized. The parasite was first named as *Schyzotrypanum cruzi* in honor of Oswaldo Cruz, but it was subsequently renamed *Trypanosoma cruzi*.

Chagas returned to Lassance looking for the presence of vertebrate hosts of this newly discovered parasite. After several tests in humans and animals, he found a cat with parasites in the bloodstream. A few weeks later, he was asked to investigate the possibility of an acute malarial episode in a two-year-old girl named Berenice living in the same house where the cat was found. He had clinically examined this girl before and now parasites were detected in the blood, suggesting the acute phase of a new disease. Further examinations demonstrated that the flagellates disappeared as the symptoms vanished, thus raising the possibility of a chronic phase of the new disease. On April 15, 1909, Oswaldo Cruz reported Chagas's discovery to the Brazilian National Academy of Medicine. A complete study on the evolutive cycle of the *T. cruzi* was published in the first volume of the Maguinhos Institute journal, *Memórias do Instituto Oswaldo Cruz*, in August 1909 [20]. On October 26 of the same year, Chagas presented his first lecture on American trypanosomiasis in the Brazilian National Academy of Medicine, calling his discovery “a new realm in Pathology”. The genius of Carlos Chagas enabled him to describe the agent, vectors, and main mechanism of disease transmission, clinical signs in humans and animals, and the existence of animal reservoirs. The merit of his research, the circumstances surrounding it, and the subsequent development of the field represent one of the most important pages in the history of medical science. Never before or since has a single scientist, a clinician and clinical investigator, fully characterized a new disease in all its aspects in this manner.

After some time, this important discovery by Chagas resulted in violent arguments and also led to denials, probably because it was beyond the comprehension of many of the physicians and scientists of those days and because his contemporaries envied him. In 1915, the campaign directed against Chagas intensified when Kraus, a German bacteriologist from the Buenos Aires Bacteriology Institute in Argentina, could not find any human cases of Chagas disease in northern Argentina, although a great number of infected bugs were found in hut dwellings. Chagas rejected Kraus's report, but the attacks and doubts against his discovery continued, and he was called “a man who searches in the jungle for diseases which do not exist”. After 1920, Chagas disease was simply forgotten and disappeared from nosology as an infectious disease of public health importance. The rediscovery of Chagas disease was made by Salvador Mazza, an Argentine physician, in 1934, just before Chagas's death, on November 8 of that year. He reported many acute cases found in northern Argentina, exactly where Kraus failed to find any human case of the disease. Like Chagas, Mazza was criticized for “discovering new diseases instead of treating the many already existing ones”. Due to Mazza's research, investigations on once forgotten Chagas disease were reinstated in South America, now recognized just as a cardiopathy. Only two decades later, the now well-known late manifestations of Chagas disease mega-syndromes, usually megaesophagus and megacolon, conjectured by Chagas [21], in which the pathogenic mechanism is the intrinsic denervation of the viscera, were only recognized as of chagasic etiology through the works of Köberle [22], an Austrian-born pathologist, founder of the Department of Pathology of the Faculty of Medicine of Ribeirão Preto, University of São Paulo. In his thesis for the position of full professor, Köberle states that “… The knowledge of the high incidence of Chagas disease in Ribeirão Preto and surroundings, associated with the verification of a large number of megas in the same region, led us to suspect of the chagasic etiology of the megas in Brazil and to study its pathogenic mechanism, particularly that of the megaesophagus ….”

## Pathology of Chronic Chagas Cardiomyopathy

By the early 1990s, the World Health Organization (WHO) considered Chagas disease the most serious parasitic disease in Latin America [23] and as having the greatest economic impact. The number of estimated infected people was approximately 18 million, with a further 100 million under risk. Now, the revised numbers are much reduced, with an estimate of about 10–13 million [24] or, even less, 8–10 million infected people [25], [26]. Large-scale local initiatives to halt vector-borne transmission together with the improvement of blood-donor screening tests to control blood transfusion and congenital *T. cruzi* infection transmission, such as the “Southern Cone Initiative”, explain most of this success [27]. Notwithstanding, Chagas disease is still classified as one of the most neglected diseases in the world [28], since there are still 200,000 new cases of Chagas disease notified each year and some rural communities in Latin America with seroprevalence rates as high as 40% [29]. Although primary infection continues to endanger the lives of countless people in Latin America, the real challenge concerning the millions of chronic chagasic patients is the control and treatment of the chronic manifestations of the disease. For this it is essential to understand the pathogenesis of the late manifestations of the disease.

Chagas disease is characterized by three phases: acute, indeterminate or latent, and chronic. The heart is the most severely and frequently involved organ. The cardiac involvement during the acute phase varies from mild (asymptomatic or olygosymptomatic) to severe. The latter may be fatal, occurring in 3%–5% of cases. The indeterminate or latent phase, between the acute and chronic phases, usually of long duration (up to 10–30 years), is characterized by the absence of clinicopathological evidence and is usually accompanied by either a normal electrocardiogram or one with minor disturbances of cardiac rhythm. Approximately 30% of the infected individuals eventually develop late manifestations. The symptomatic disease affects the heart in 94.5% of patients that are considered to have CCC, usually between 15 and 50 years of age. Congestive heart failure is the cause of death in 58% of these patients, whereas cardiac arrhythmias and unexpected death affects 36.5%. The remaining manifests as mega-syndromes of hollow viscera, usually megaesophagus and megacolon.

Three stages are seen in CCC. In the initial stage the patient has a few symptoms, usually related to disturbances of rhythm. In the intermediate stage the clinical signs usually correlate with a mildly to moderately enlarged heart. In the final stage, the most significant clinical manifestation includes congestive heart failure, thromboembolic phenomena, severe arrhythmias, and sudden death [11], [30]–[33]. Alterations in cardiac function, severe conduction abnormalities, and episodes of ventricular arrhythmias or syncope are considered to predispose chagasic patients to sudden unexpected death, a significant risk at any stage of the disease [11], [33], [34].

**Figure 1:**
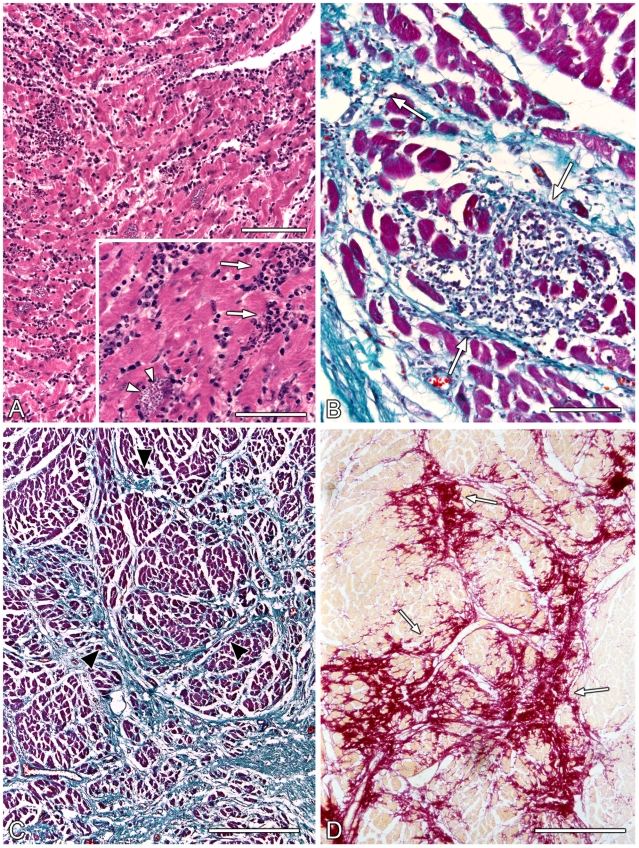
Micropathology of Chagas heart disease. (A) Acute myocarditis with foci of myocytolytic necrosis and degeneration are seen with an intense inflammatory infiltrate around ruptured pseudocysts of parasite (arrows, in the inset). Intact intramyocyte parasite nest without inflammatory response (arrow heads, in the inset). Hematoxylin and eosin staining. Bar = 100 µm; inset bar = 50 µm. (B) Chronic fibrosing myocarditis. Foci of myocytolytic necrosis associated with mononuclear inflammatory infiltrate and incipient interstitial fibrosis appearing in light blue (arrows). Gomori trichrome staining. Bar = 100 µm. (C) Chronic fibrosing myocarditis. Predominantly perimysial interstitial fibrosis extending to the endomysium (arrow heads) appearing in light blue associated with mononuclear inflammatory infiltrate. Gomori trichrome staining. Bar = 500 µm. (D) Chronic fibrosing myocarditis. Interstitial and diffuse fibrosis manifested by increased amount of thick collagen fibers surrounding muscle fiber bundles (perimysial matrix) and around intramural coronary vessels, combined with a less pronounced increase in the endomysial matrix. Picrosirius red staining. Bar = 500 µm.

**Figure 2:**
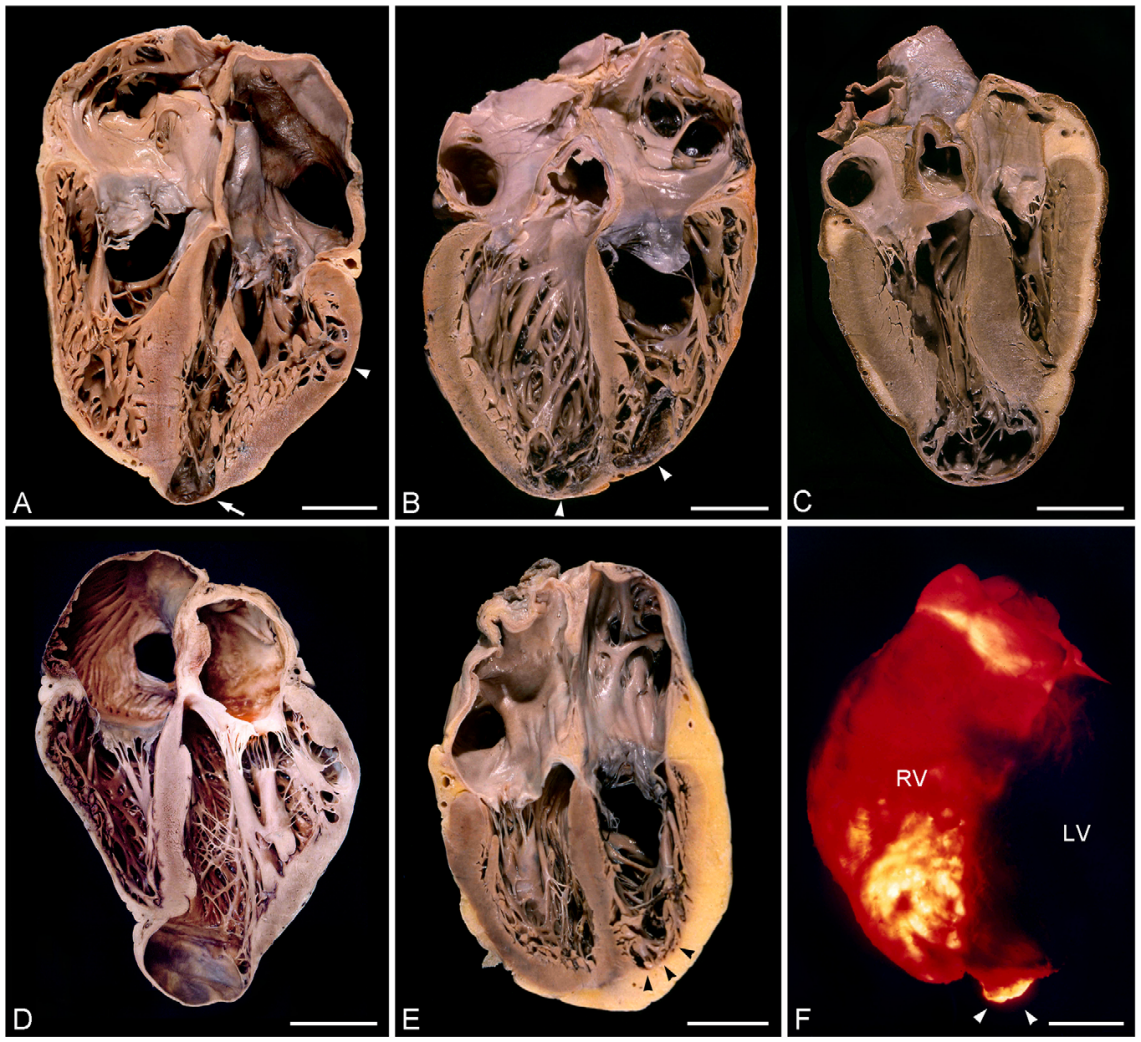
Gross pathology of chronic Chagas cardiomyopathy (four-chamber frontal view). (A) Cardiomegaly with a left apical aneurysm (arrow). Myocardium hypertrophy. Marked thinning can be noted in the obtuse border of the heart at the submitral area (arrow head). At the apex of the right ventricle, distinct replacement of myocardial tissue by adipose tissue can be seen. (B) Cardiomegaly. Thinning and thrombosis at apices of both ventricles (arrow heads). Dilatation of cardiac ventricular chambers, mainly the right one. Fibrofatty substitution at the apex of the left ventricle and major part of the right ventricular free wall. (C) Normal-sized heart showing an enormous aneurysm at the apex of the left ventricle. Hypertrophy of the right ventricle free wall except for a marked thinned apex can be clearly seen. (D) Mildly enlarged heart showing dilatation of the four chambers. Giant left apical aneurysm. Thinning of left border of the heart immediately below the mitral valve. (E) Globally enlarged chronic chagasic heart with dilatation mainly affecting the right-sided chambers. Adipose replacement of the right ventricular myocardium, particularly at the apical region, associated with bulging can be seen (arrow heads). (F) Transillumination of a chagasic heart showing thinning of the muscle wall “cor bifidum” with aneurysm at the left apex (arrow heads), and marked thinning of the anteroapical region of the right ventricle. RV, right ventricle; LF, left ventricle. All bars = 3 cm.

The main pathological changes reflect the importance of the involvement of the heart in Chagas disease [11], [18], [30], [33], [35]. In the acute phase, the heart is globular and flabby. Foci of myocytolytic necrosis and degeneration are observed microscopically with an intense mononuclear inflammatory infiltrate and intense parasitism of myofibers (Figure 1A). Most of the hearts with CCC show a marked alteration in size and form, although some hearts appear to be normal in size and form (Figure 2). All degrees of enlargement of the heart may be found, mainly affecting the right-sided chambers of the heart, with dilatation being more pronounced than hypertrophy. More than 50% of chagasic hearts show a peculiar lesion of the apex of the heart, mainly the left ventricle apex, consisting of thinning and bulging of the apical region, the so-called apical aneurysm. Similar localized parietal thinning may occur in the left and right ventricular free walls. Fibroadipose or adipose replacement of the right ventricular myocardium, particularly at the apical area of the right ventricle, may be observed, occasionally associated with a bulging of the apex. This attenuation of the myocardium implicates mainly the apical region, although the entire right ventricular free wall can be involved. Thrombosis of the aneurysm is common. Even without aneurysm, extensive mural thombosis in the lower part of the left ventricle and in the dilated right auricle may be seen [36]. The presence of thrombi explains the frequent occurrence of thromboembolic phenomena in the pulmonary and systemic circulation. In CCC, micropathology reveals focal and diffuse chronic fibrosing myocarditis (Figure 1C and 1D). Diffuse foci of myocardial micronecrosis are present and associated with an inflammatory infiltrate composed predominantly of lymphomononuclear cells and interstitial fibrosis, one of the most prominent features (Figure 1B) [37]–[40]. This remodeling of the collagenous matrix leads to progressive myocardial decompensation by decreased cardiac output, combined with an increased workload due to myocardial stiffness. The conduction system shows inflammatory and fibrotic lesions similar to those found in the myocardium [34], [41]. Myofibers containing parasites are virtually never found in the chronic phase of the disease. Destruction of the intrinsic cardiac and enteric nervous system (chiefly parasympathetic ganglion cells) and mediastinal paraganglia has been demonstrated [11], [42], [43].

## Theories on the Pathogenesis of CCC

The pathogenesis of chronic chagasic cardiopathy is still not fully understood. Different mechanisms have been proposed.

### Direct Tissue Destruction by *Trypanosoma cruzi*

The existence of different clinical forms of the disease was soon identified, which was at first thought to be associated with differences in the parasites implicated. Indeed, even Chagas noticed a peculiar dimorphism, the so-called slender and stout forms of the parasite in the bloodstream, observations later confirmed by many others [44], [45]. Today, these two morphological forms are believed to emerge from epigenetic phenomena and their pathological relevance is obscure [46]. At a very early stage, the idea of a major role of differential tissue tropism in the pathogenesis of Chagas disease was proposed [47], [48]. This idea has persisted in spite of only tenuous evidence based mainly on the parasite distribution in different tissues in the acute phase of experimentally infected animals [49]–[51].

In chronic Chagas disease, parasites are rarely found in tissues examined by routine techniques [11], [52]. However, parasite antigens were disclosed in the myocardial tissue by application of immunohistochemical techniques [53] and sensitive polymerase chain reaction (PCR) [54]–[56]. These observations support a role for persistent antigenic stimulation throughout the chronic phase in the pathogenesis of myocardial changes. New studies may highlight the primary role of *T. cruzi* in the pathogenesis of Chagas disease and set the stage for establishing the notion that genomic variation of *T. cruzi* might influence the course of the disease.

### Autonomic Abnormalities

The autonomic nervous system of patients with Chagas disease has been extensively studied [11], [13]. CCC could be a neurogenic form of heart disease promoted by the destruction of the parasympathetic ganglions cells in the heart. Early morphological investigations revealed a conspicuous reduction in the number of cardiac parasympathetic neurons of patients who had died from intractable congestive heart failure. The extent of heart denervation seen in Chagas disease has not been detected in any other cardiopathy so far studied, though a number of cardioneuropathies have been described [57]. Abnormalities of autonomic heart rate control were also described in clinical studies of asymptomatic patients with cardiac enlargement on chest X-rays [58], [59]. Malignant ventricular tachyarrhythmias (ventricular tachycardia, ventricular fibrillation) are major causes of sudden death among patients with CCC. The destruction of the parasympathetic innervation could induce an increased sympathetic tone with either a direct effect in arrhythmogenesis via altering the electrophysiologic properties of the heart or an indirect effect via other mechanisms, such as increased oxygen demand by catecholamines, increased coronary vasomotor tone, and augmented platelet adhesiveness [60]. It is now well established that the intrinsic denervation of organs occurs in the acute phase of the disease because of IFN-γ-elicited nitric oxide (NO) production resulting from inducible nitric oxide synthase (iNOS) activation of the inflammatory foci [43]. The main dilemma of the neurogenic theory remains in the uncertainty about its physiopathologic mechanism, i.e., its implication in the pathogenesis of the chronic fibrosing myocarditis.

### Role of Autoimmune Mechanisms

The participation of autoimmune mechanisms in the genesis of the chronic myocarditis of Chagas disease has been postulated [14]–[16]. The relative lack of parasites in the myocardium during the chronic phase was the origin of many autoimmune theories, including both a humoral and cellular origin. The establishment of an organ-specific autoimmune nature for Chagas disease chronic fibrosing myocarditis has been waiting on an experimental model that could provide evidence in support of the hypothesis and allow specific manipulations by which different sets of lymphocytes could be implicated in the generation of the disease. Perhaps the most compelling evidence supporting the role for autoimmunity comes from the demonstration that anti-CD4 abrogates rejection and reestablishes long-term tolerance to syngeneic newborn hearts grafted in mice chronically infected with *T. cruzi* [61], although this does not happen when different strains of parasites and mice are employed [62].

Two criticisms are often used against the hypothesis that CCC is an autoimmune disease. The first is that immunosuppressants, which generally relieve symptoms of autoimmune diseases, exacerbate mortality in individuals with Chagas disease, and the second is that therapy directed at the parasite often ameliorates the clinical disease in humans and experimental animals [63].

It has been assumed that autoimmunity is triggered after the initial contact with the parasite and that immunological processes continue during the chronic phase of the disease [64]. This, however, has not been proven. Most of the inference on the alleged immunological mechanisms implicated in the chronic phase of cardiac disease pathogenesis, when rarely a parasitic pseudocyst can be detected, is based on experiments with animals acutely infected with *T. cruzi* in which the acute myocarditis is directly related to the presence of the parasite.

### Studies Involving Microcirculation and Chagas Disease

#### Studies in mice and rats

**Figure 3:**
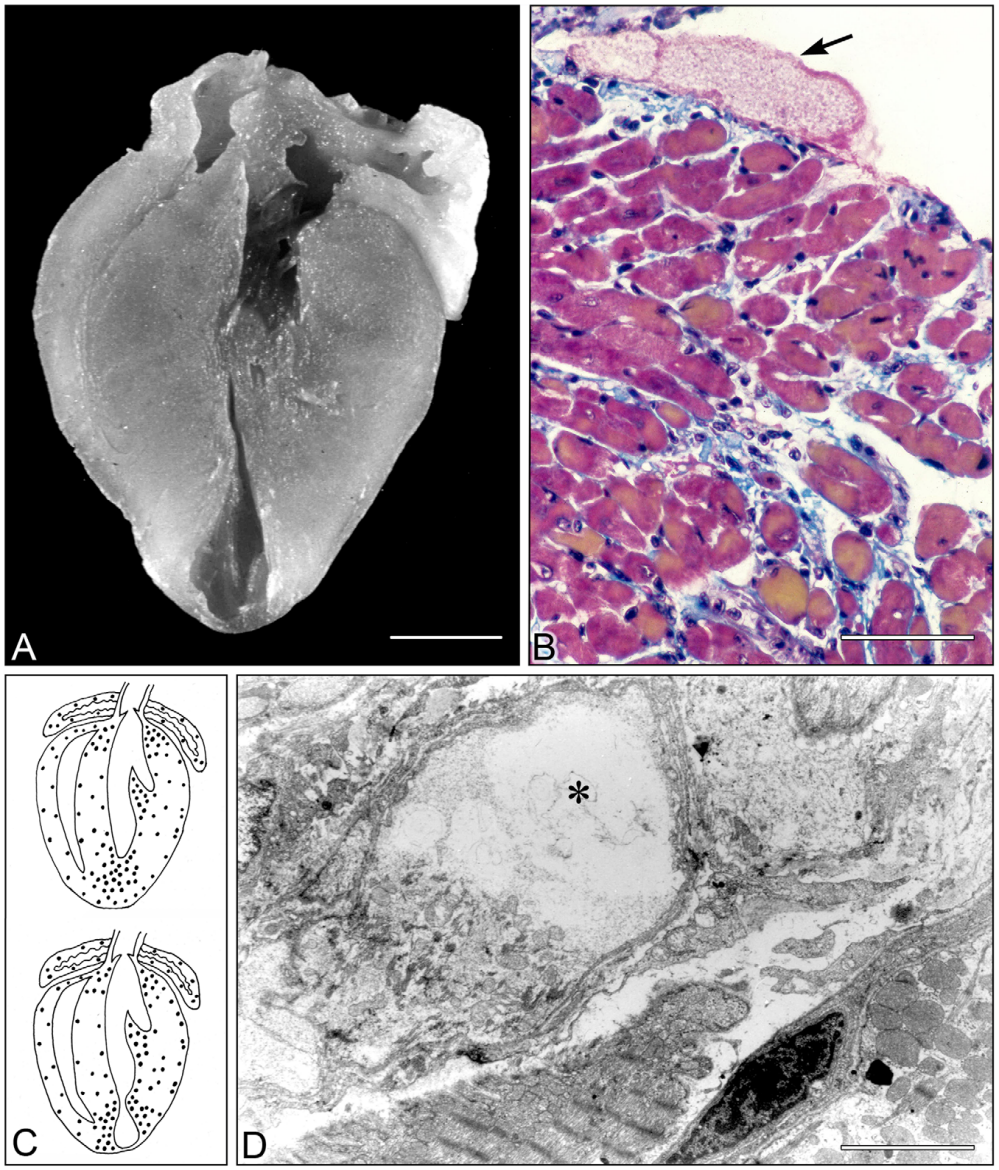
Study in mice chronically infected with *T. cruzi* demonstrating involvement of microcirculation. (A) Enlarged heart of a mouse infected with *T. cruzi* 100 days post-infection showing marked thinning of the apex of the left ventricle (apical aneurism). Bar = 2 mm. (B) Myocardium of an infected mouse stained by the Carstairs method for demonstration of platelets. An occlusive platelet thrombus is seen in a small epicardial vessel (arrowhead). Bar = 50 µm. Mononuclear cell infiltration, interstitial edema and fibrosis, and foci of myocytolytic necrosis. (C) Schematic representation of coronal sections through mice hearts infected with *T. cruzi* 100 days post-infection without (upper panel) and with (lower panel) apical aneurism, showing the extent of foci of myocytolytic necrosis. These areas are scattered throughout the ventricular and atrial myocardium, but are more numerous in the subendocardial and subepicardial regions in the apex, papillary muscles, and base of the ventricles. (D) Electron micrograph showing complete dissolution of myofibrils within a myofiber (*) of an infected mouse with characteristic myocytolysis or myocytolytic necrosis. Bar = 10 µm.

In mice first immunized with several inoculations with epimastigote forms of the avirulent PF strain of *T. cruzi* and then challenged with trypomastigotes of the virulent Colombian strain of *T. cruzi*, isogenic BALB/c mice developed a cardiomyopathy very similar to that observed in the chronic phase of human Chagas disease [7]. Macroscopically, there was cardiomegaly with hypertrophy and dilatation of the ventricular chambers associated with thinning of the left ventricle apex in 46% of the hearts (apical aneurysm) (Figure 3A). The microscopic findings revealed focal areas of myocytolytic necrosis and myocardial degeneration associated with a lymphomononuclear inflammatory infiltrate accompanied by interstitial fibrosis and occasional pseudocysts. In addition, platelet aggregates, forming transient occlusive thrombi, were detected in small epicardial and intramyocardial vessels, direct evidence of microcirculatory disease (Figures 3B and 3D). Moreover, the focal nature of the myocardial lesion and the type of myonecrosis represent the indirect evidence for the involvement of the microcirculation in this model (Figure 3C). The release of vasoconstrictor substances, such as thromboxane A2 (TXA_2_) and platelet activating factor (PAF) by macrophages, which are the predominant inflammatory cells, was proposed to cause transient ischemia and myocytolytic necrosis [8].

**Figure 4:**
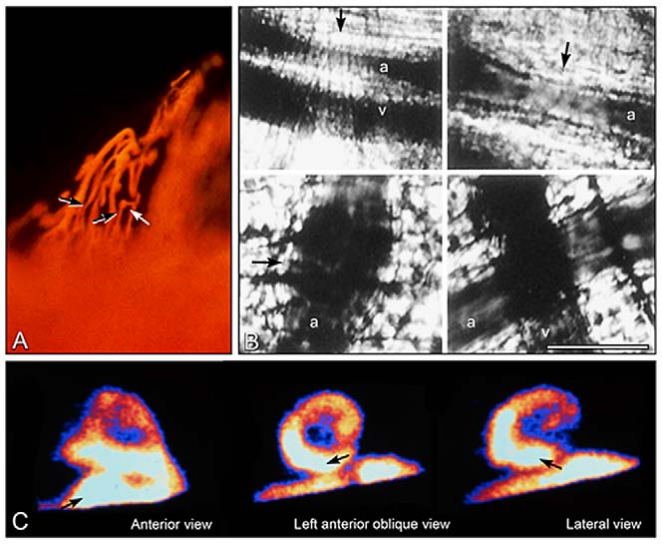
Changes of coronary perfusion in experimental *T. cruzi* infection and human chronic Chagas cardiomyopathy. (A) Microfil injection of the coronary vasculature of A/J mice infected with *T. cruzi* 15–17 days post-infection. Section of the atrium reveals saccular microaneurysms and vasospasm in the subendocardium. (B) Videomicrographs of representative fields of the microvasculature obtained from the cremaster muscle from *T. cruz*i–infected mice 20–25 days post-infection (a, arterioles; v, venules). Upper left and right panels: Representative fields showing areas of vasospasm (arrows). Left lower panel: In this field there is an area of segmental microvascular dilation (arrow). Right lower panel: Infected mouse treated with verapamil in which there were no areas of vasospasm or dilatation. Bar = 20 µm (from Tanowitz et al. (1996) Journal of Parasitology 82: 124–130, with permission of Allen Press and the Journal). (C) Planar images (anterior, left anterior oblique, and lateral views of myocardial scintigraphy with Tc-labelled microspheres in a chronic chagasic patient whose complaint was chest pain, but who had angiographycally normal coronary arteries. A prominent perfusion defect is seen in the anterolateral and posterolateral regions of the left ventricle. Courtesy of J. Antonio Marin-Neto, MD.

A/J mice infected with the Brazil strain and perfused with silicone rubber (Microfil) 15–17 days post-infection revealed numerous areas of focal vascular constriction, microaneurysm formation, dilatation, and proliferation of microvessels (Figure 4A), which is similar to the results described for other congestive cardiomyopathies [4]. These microvascular changes, observed prior to the onset of significant myocardial degeneration or fibrosis, were reduced to a minimum by long-term administration of verapamil [65]. These observations were corroborated by direct in vivo visualization utilizing a surrogate murine model, i.e., the cremaster microvascular bed [66]. Direct observation of the effects of *T. cruzi* infection on microcirculatory flow in vivo and quantitative measurement of parameters like velocity of red blood cell flow (Vrbc) and vessel diameter were provided. When the cremaster model was examined 20–25 days post-infection in male CD-1 mice infected with the Brazil strain, a significant decrease in Vrbc, reversed by verapamil treatment, was observed in the first- and third-order arterioles and venules. In addition, the marked inflammatory response attenuated was by verapamil treatment. The arterioles of the infected mice exhibited segmental areas of vasospasm and dilatation, possibly the initiating event in microaneurysm formation (Figure 4B) [67].

The exact mechanism of such vascular lesions has not been fully clarified. In addition to spastic phenomena, the observation of platelet thrombi in the coronary microcirculation of infected mice led to an investigation of the possible mechanisms involved. Toward this end, a study using A/J mice and human umbilical vein endothelial cells (HUVECs) infected with the Tulahuen strain of *T. cruzi* showed an increased aggregation of platelets during the early stage, a factor that may contribute to the development of thrombosis. In addition, increased levels of TXA_2_ were observed. This finding could contribute to the increased intravascular platelet aggregation and focal microvascular spasm [68]. Recently, Ashton and colleagues [69] demonstrated that all three life forms of the parasite are capable of synthesizing TXA_2_, but it was most dramatic in amastigotes. These observations suggest that TXA_2_ could contribute to the pathogenesis of CCC and its clinical manifestations.

Early in the course of infection, parasites are evident in the coronary microvascular endothelial cells (ECs) before parasitemia can be detected, suggesting that the coronary endothelium could be an initial, if not primary, target of *T. cruzi* infection. Acutely infected rats developed changes in the endothelial layer characterized by EC swelling and a few points of cytoplasmic discontinuity that appeared as holes exposing the subendothelial collagen that is usually associated with platelet-fibrin aggregates, which might affect the generation of vasoactive substances, and impairs the equilibrium between opposing forces [70]. In vitro and in vivo studies indicate that infection of the endothelium results in expression of both pro-inflammatory cytokines and vascular adhesion molecules, which are both important components of the inflammatory response [71]–[73] *T. cruzi* infection of ECs was demonstrated to cause activation of the NF-κB pathway, likely contributing to the induction of cytokine and adhesion molecular expression in the endothelium [72]. Furthermore, in the myocardium obtained from *T. cruzi*–infected humans and experimental animals, increased expression of cytokines, iNOS, and adhesion molecules has been reported [73]–[75].

Endothelial cells are the major source of endothelin 1 (ET-1), a potent vasoconstrictor, and its role in the pathogenesis of chagasic heart disease has been demonstrated. The infection of CD1 mice with the Brazil strain and C57BL/6 mice with the Tulahuen strain caused an intense vasculitis, high plasma ET-1 levels, and increased expression of mRNAs for the precursor molecule preproET-1, endothelin converting enzyme (ECE), and ET-1 in the myocardium [76]. It has been hypothesized that *T. cruzi*–derived molecules provoke overexpression of ET-1 [77]. Elevated levels of plasmatic ET-1 have been demonstrated in patients with CCC [78]. To further test the hypothesis that ET-1 contributes to the pathogenesis of murine chagasic cardiomyopathy, mice with a deletion of the gene for ET-1 in either cardiomyocytes or ECs were used to distinguish between ET-1 derived from both cell types. In infected mice in which the gene for ET-1 was deleted in cardiomyocytes, there was a reduction in myocardial inflammation and fibrosis [79]. In addition, these mice displayed a reduction in cardiac enlargement as revealed by cardiac magnetic resonance imaging and echocardiography. This data provided further evidence of a role for ET-1, particularly myocyte-derived ET-1, in the pathogenesis of CCC.

#### Studies in dogs

Hearts from dogs sacrificed 18 to 26 days after intraperitoneal inoculation with trypomastigote forms of the 12SF strain of *T. cruzi*/kg body weight were studied. This study demonstrated that myocarditis characterized by small focal areas of lesion and myocytic necrosis associated with interstitial mononuclear infiltration. Ultrastructurally, degenerative changes were observed in ECs in contact with T lymphocytes, as well as platelet aggregates and fibrin thrombi in the intramyocardial capillaries. These alterations suggested that a possible interaction between ECs and effector immune cells might play an important role in the pathogenesis of the myocellular lesion and of the microangiopathy observed in this model [80].

#### In vitro studies

Direct infection of human endothelial cells in culture with *T. cruzi* resulted in the alteration of various critical biochemical processes responsible for the maintenance of microvascular perfusion, such as Ca^2+^ homeostasis and generation of inositol trisphosphate and prostaglandin I_2_ [81], [82]. *T. cruzi* infection of HUVECs results in an alteration of cyclic AMP metabolism, which plays a protective role against the direct and/or indirect lesion caused by the adhesion and aggregation of circulating platelets to ECs [83]. However, inflammatory cells may contribute to a state of microvascular hypoperfusion by secreting cytokines and other factors known to affect platelets and ECs.

Cytokines contribute to the pathogenesis of various parasitic infections, and their roles in the pathogenesis of *T. cruzi* infection have been extensively studied. The increase of interleukin-1β (IL-1β), IL-6, and colony stimulating factor 1 (CSF-1) in infected ECs may lead to alterations in their function [83]. IL-1β is elaborated by activated macrophages and by peripheral blood mononuclear cells, including those infected with *T. cruzi*, and by a variety of other cell types, such as ECs [84], [85]. The antithrombotic properties of ECs may be altered by IL-1β. This cytokine may reduce tissue production of the plasminogen activator and increase production of the inhibitor of this activator, an event that may result in thrombus formation [86], [87]. Although the products of IL-6 are markedly increased in EC cultures, it has not been possible to determine whether non-infected cells are also induced to produce this cytokine. Since IL-1β may induce IL-6 production by ECs, it is not clear whether IL-6 production by infected cells is a direct result of the infection or is induced by the IL-1β produced in response to infection. CSF-1 is an important growth factor for the proliferation and maturation of cells of the mononuclear lineage [85]. It is also important in recruitment, possibly acting in conjunction with IL-1β. High CSF-1 levels have been detected in cultured ECs infected with *T. cruzi*. These observations may reflect the growth of the monocyte population in the microvasculature, resulting in the later elaboration of proinflammatory cytokines [71]. In addition, trypomastigotes may elaborate a neuraminidase that may be involved in the removal of sialic acid from the surface of mammalian myocardial cells and ECs, facilitating thrombin binding. The loss of this endothelial surface protector molecule may contribute to platelet aggregation and thrombosis within the small coronary vessels [88]. These factors acting together may ultimately result in spasm and thrombosis in the small coronary vessels, inducing focal myocardial damage.

The consequences of *T. cruzi* infection of HUVECs with regard to the production of biologically active ET-1 are an increased expression of ET-1 mRNA [89]. Increased production of ET-1 may contribute to the coronary microvascular vasoconstriction previously reported in experimental Chagas disease [9].

#### Studies in humans

Anatomical studies have shown structural derangement and rarefied microvasculature in the left ventricular myocardium. A histotopographical study comparing the microcirculatory system after injection of an opaque medium into chagasic and control human hearts demonstrated focal decapillarization in chronic Chagas disease due to extraluminal compression, suggesting that this might be the cause of focal myocytolytic necrosis [90]. Similarly, a postmortem radiological study of chagasic hearts revealed vascular changes at the heart apex characterized by distorted and/or scarce vessels associated with decreased arterial density, presumably related to the pathogenesis of apical aneurysm [91].

The evaluation of chest pain is a major problem in chagasic patients. Almost all exhibit symptoms that are atypical for classic angina pectoris. Although symptoms suggestive of myocardial ischemia are present, coronary angiographical studies show normal or nearly normal coronary arteries in more than 90% of patients studied [92]. This peculiarity had been previously reported in a postmortem study [93]. However, patients specifically selected on the basis of chest pain did show perfusion abnormalities detectable by thallium-201 scintigraphy, suggesting that myocardial ischemia, possibly of the microvascular type, may contribute to the genesis of the symptoms (Figure 4C).

Abnormal perfusion in different groups of chagasic patients has been confirmed by various independent investigators using isonitrile-99m-technetium [94] or thallium-201 [92], [95]. In addition, myocardial capillary blood flow in chronic chagasic patients with no significant clinical or electrocardiographic manifestations proved to be markedly reduced when evaluated with rubidium-86, while the major coronary vessels appeared normal. The reduction observed, comparable to that exhibited by a group of non-chagasic patients with obstructive coronary disease, occurred under basal conditions [96] and, to a lesser extent, during exercise [97]. Using a specific marker of regional flow independent of cell metabolic activity, a perfusion defect was detected in 55% of the 18 chagasic patients with CCC and essentially normal epicardial coronary circulation [98].

Vasospastic mechanisms have been proposed in the genesis of coronary accidents in patients with CCC [99]. For example, it was demonstrated that cardiopathic chagasic patients present an abnormal, endothelium-dependent, coronary vasodilating mechanism as demonstrated by acetylcholine and adenosine infusion into the left coronary artery, suggesting that epicardial and microvascular coronary reactivity may be altered in these patients. The clinical importance of this alteration awaits elucidation. However, this abnormality of the coronary microvasculature may contribute to the genesis of the symptoms related to the ischemic processes observed in chronic chagasic patients and to acute myocardial infarction in the absence of significant coronary damage [100].

Biopsies obtained from chronic chagasic hearts revealed a marked thickening of the basement membrane in most myocytes and capillaries (up to 20 times the normal thickness) [101]. This alteration is similar to the thickening with or without multiple layers reported for the basement membranes of myocardial capillaries in other cardiomyopathies [102]. A very well-developed capillary network has been observed in chagasic human hearts using a cell-maceration scanning electron microscopic method [103]. This alteration may represent the probable cause of slow capillary flow, contributing to the hypoxic changes observed in CCC.

Significant dilatations of arterioles and capillaries in various ventricular areas of chagasic hearts compared to hearts with dilated cardiomyopathy were described. It was hypothesized that such microcirculatory dilatations could cause inadequate blood flow distribution in the watershed area lying between the two main coronary flow sources (the anterior descending and posterior descending arteries, and the right and circumflex coronary arteries), resulting in ischemic and extensive fibrosis within the left ventricle apical and posterior regions [104].

The relation of regional sympathetic denervation and myocardial perfusion disturbance to wall motion impairment was described in patients with CCC. Global left ventricular function, segmental wall motion analysis, and myocardial perfusion were evaluated in 58 patients, demonstrating myocardial perfusion defects in the absence of epicardial coronary artery disease. In addition, the extension and severity of perfusion abnormalities parallel the progression of myocardial damage. These results support the notion that perfusion disturbance in CCC may be caused by transient disturbances of coronary blood flow regulation at the microvascular level [105]. The same group correlated the clinical, electrocardiographic, angiographic, electrophysiologic, and wall motion/myocardial perfusion disturbances in chronic chagasic patients with either sustained or non-sustained ventricular tachycardia. The fact that both fixed perfusion defects (reflecting local fibrosis) and reversible and paradoxical defects predominate in the dyssynergic arrhythmogenic left ventricle region is also compatible with the hypothesis that microvascular ischemia plays an important role. Thus, several findings suggest that transient disturbances of coronary blood flow regulation at the microvascular level may be a causative mechanism of regional myocardial degeneration, with a consequent reparative fibrosis that ultimately constitutes the substrate for reentrant circuits and the appearance of both sustained and non-sustained ventricular tachycardia [106].

### Chronic Chagas Heart Disease and the Interstitial Matrix

In CCC there is extensive damage of the myocardium and, consequently, it is not surprising that interstitial fibrosis is one of the most prominent features [38]. Since the extracellular matrix has an important role in the structure and function of the myocardium [107]–[109], the progressive accumulation of interstitial collagen could well be the main factor responsible for the progressive impairment of the contractile performance of the myocardium and for the increase in arrhythmogenic risk in chronic Chagas heart disease.

The pattern of myocardial fibrosis in chronic Chagas heart disease probably reflects the pathogenic mechanisms involved. Diffuse foci of myocardial myonecrosis may be the main etiology factor of the chronic expression in chronic chagasic cardiomyopathy [110]. The presence of infiltrates of lymphomononuclear cells is a consistent and prominent finding in the chronic chagasic fibrosing myocarditis. Our results clearly showed the colocalization of the fibrosed areas and fibroblasts with T lymphocytes and macrophages. T lymphocytes have been demonstrated to play a role in the pathogenesis of fibrosis. Bleomycin-induced pulmonary fibrosis in mice is attenuated by depletion of CD4^+^ or CD8^+^ T cells and completely abrogated by total T cell depletion [111]. CD4^+^ and CD8^+^ cells may act directly on mesenchymal cells by means of cytokine production that leads to the proliferation of fibroblasts and the synthesis of collagen or indirectly by enhancing the activation of macrophages [111]. Macrophages, when activated by cytokines, have been shown to produce powerful inducers of fibrosis, such as transforming growth factor β (TGF-β) and platelet-derived growth factor (PDGF) [112], [113]. Our study shows a predominance of T cells and macrophages concentration. Besides, since myocytes can produce growth factors such as fibroblast growth factor [114], the injured myocytes could potentially produce and release such factors, contributing to fibrogenesis. In addition, increased production of ET-1 by cardiac myocytes correlates closely with the degree of hemodynamic and functional impairment [76], [115], indicating that this peptide could also contribute to myocardial fibrosis through its collagen synthesis-enhancing effect [116].

### Studies on Therapeutic Strategies

Increasing evidence for abnormalities at the microvascular level has been accumulated from studies both in chagasic patients and experimentally infected animals. The changes suggest that myocardial lesions develop, at least in part, as a consequence of additive and progressive cell necrosis initiated and perpetuated by changes in myocardial microcirculation. Based on these studies, it is possible to speculate that therapy aimed at decreasing the degree of microvascular ischemia could prevent or ameliorate myocardial damage, ventricular dysfunction, and ventricular arrhythmias.

Angiotensin-converting enzyme inhibitors (ACEIs) have emerged as the treatment of choice for patients with all degrees of heart failure, ranging from asymptomatic left ventricular dysfunction to severe heart failure. The mechanism of action of captopril, an ACEI, involves suppression of angiotensin II, a potent vasoconstrictor, and increased levels of bradykinin through the inhibition of kininase II, which induces NO release in ECs and stimulates the production of prostacyclin, a vasodilating prostaglandin [117]. Captopril is commonly given to patients with CCC. Despite routine administration of captopril to patients with CCC, few studies have examined the effects of this drug in these individuals. Captopril has been shown to improve cardiac function with few side effects [118], [119] but has not been found to reduce mortality [120]. In a study using A/J mice infected with a Brazil strain of *T. cruzi*, the animals developed acute myocarditis 21 days after infection, characterized by severe focal inflammation, necrosis, and fibrosis. The administration of captopril significantly reduced necrosis and fibrosis in infected mice. Taken together, these results suggest that captopril can reduce myocarditis and fibrosis in *T. cruzi* infection.

The effect of enalapril, another ACEI, on ventricular function in patients with CCC has been evaluated. A significant improvement of diastolic function and a trend to improvement of systolic function was seen in the group that received digitalics, diuretics, and enalapril in comparison with the group that received digitalics and diuretics only. This beneficial action of enalapril in diastolic function could be related to decreased venous return and increased arteriolar vasodilatation [121].

Two cases of myocarditis due to acute Chagas disease, resulting from oral intake of sugar cane juice infected with *T. cruzi*, developed acute decompensated NYHA class IV heart failure. Both patients were refractory to treatment with ACEI, aldosterone antagonists, and dobutamine. Levosimendan was prescribed, and clinical improvement was observed, with progression to NYHA class II. Levosimendan is a positive inotropic drug with vasodilatory properties with anti-ischemic action through increased coronary flow and reduced preload and afterload. The limitations of this study include lack of studies with a placebo group to ensure that the benefits are a result of the drug and not side effects of other medications [122].

Further evidence of the microvascular involvement in CCC pathogenesis has been demonstrated by studies with verapamil, a first generation L-type calcium channel antagonist [123]. *T. cruzi*–infected CD1 mice given verapamil immediately after infection showed decreased mortality and attenuated myocardial inflammation and fibrosis [65], [67]. However, when verapamil was administered after 60 days post-infection, there was no amelioration of infection-associated structural and functional abnormalities. The results suggested that verapamil acts early in the course of *T. cruzi* infection to prevent ventricular dilatation and myocardial dysfunction [124]. Verapamil has several well-known actions that may reduce the severity of cardiomyopathy, including increasing coronary blood flow, inhibition of calcium channels and α and β-adrenergic activity, and platelet aggregation [123].

Phosphoramidon, a potent inhibitor of endothelin-converting enzyme, reduced myocardium inflammation and fibrosis and attenuated right ventricle diameter increase in CD1 mice infected with the Brazil strain of *T. cruzi* and treated for the initial 15 days post-infection [125].

## Conclusions

The pathogenesis of chronic chagasic cardiomyopathy, which takes decades to develop after the initial infection with *T. cruzi*, occurs as a consequence of several physiopathological processes. The very rare finding of parasites in the myocardium in the chronic phase of the disease is out of proportion to the degree of organ compromise and dysfunction. The chronic fibrosing myocarditis development is related to progressive and additive focal cellular necrosis and associated with inflammatory mononuclear infiltrate and reactive and reparative interstitial fibrosis and surrounding myocytes hypertrophy. Based on the evidence presented in the present review, these processes may be initiated and perpetuated by alterations in the myocardial microcirculation. The intrinsic and/or extrinsic cardiac necrosis system abnormalities and immunological mechanisms may contribute, but there has been extensive debate on their significance on the marked cardiac damage.

The treatment of the chronic Chagas heart disease has relied on the same conventional treatments for other cardiomyopathies. The studies cited illustrate the potential benefit of therapeutics aimed at the underlying pathophysiological microvascular mechanism of CCC. However, the favorable effect of these changes has not yet been established due to the lack of randomized multicenter clinical trials using different treatments to determine their impact on clinical improvement and survival of chronic chagasic patients. There is an urgent need for developing adequate specific treatment procedures, particularly during the chronic phase of the disease.
